# Deficiency of skeletal muscle Agrin contributes to the pathogenesis of age-related sarcopenia in mice

**DOI:** 10.1038/s41419-024-06581-1

**Published:** 2024-03-09

**Authors:** Jie Chen, Hong Chen, Xia Dong, Tiankun Hui, Min Yan, Dongyan Ren, Suqi Zou, Shunqi Wang, Erkang Fei, Wenhua Zhang, Xinsheng Lai

**Affiliations:** 1 School of Basic Medical Sciences, Jiangxi Medical College, Nanchang University, Nanchang, 330031 Jiangxi China; 2 Institute of Biomedical Innovation, Jiangxi Medical College, Nanchang University, Nanchang, 330031 Jiangxi China; 3 School of Life Science, Nanchang University, Nanchang, 330031 Jiangxi China

**Keywords:** Experimental models of disease, Muscle stem cells

## Abstract

Sarcopenia, a progressive and prevalent neuromuscular disorder, is characterized by age-related muscle wasting and weakening. Despite its widespread occurrence, the molecular underpinnings of this disease remain poorly understood. Herein, we report that levels of Agrin, an extracellular matrix (ECM) protein critical for neuromuscular formation, were decreased with age in the skeletal muscles of mice. The conditional loss of Agrin in myogenic progenitors and satellite cells (SCs) (*Pax7* Cre:: *Agrin* flox/flox) causes premature muscle aging, manifesting a distinct sarcopenic phenotype in mice. Conversely, the elevation of a miniaturized form of Agrin in skeletal muscle through adenovirus-mediated gene transfer induces enhanced muscle capacity in aged mice. Mechanistic investigations suggest that Agrin-mediated improvement in muscle function occurs through the stimulation of Yap signaling and the concurrent upregulation of dystroglycan expression. Collectively, our findings underscore the pivotal role of Agrin in the aging process of skeletal muscles and propose Agrin as a potential therapeutic target for addressing sarcopenia.

## Introduction

Skeletal muscle, a complex and essential tissue for coordinated body movements, undergoes a gradual loss of mass and function with aging, a phenomenon commonly referred to as “sarcopenia” [1, 2]. Recently, the World Health Organization has officially recognized sarcopenia as a disease, given its high incidence rate in the elderly [3, 4]. The ramifications of muscle weakness associated with sarcopenia extend to an increased susceptibility to falls, fractures, and physical disabilities, significantly compromising the quality of life for the aging population. Notably, a specific pharmacological remedy tailored for the treatment of sarcopenia is currently lacking. Several pharmacological approaches have been proposed to alleviate the symptoms of sarcopenia, primarily revolving around hormone therapies encompassing growth hormone, pioglitazone, dehydroepiandrosterone, and testosterone [5–9]. Despite these efforts, the effectiveness of hormonal interventions remains constrained and uncertain. Moreover, they often give rise to adverse effects in the body. Therefore, there is a critical need to explore the molecular mechanisms of sarcopenia and identify novel and effective therapeutic targets.

Agrin, a extracellular heparan sulfate proteoglycan, is widely recognized for its pivotal role as a critical organizer of postsynaptic differentiation at the neuromuscular junction (NMJ) [10–12]. Agrin binds to low-density lipoprotein receptor-related protein 4 (Lrp4) to activate the receptor tyrosine kinase MuSK, subsequently triggering the aggregation of acetylcholine receptors (AChRs) [13–15]. Significantly, neuroprotease neurotrypsin facilitates the proteolytic cleavage of Agrin, triggering NMJ inactivation and instability, consequently leading to muscle wasting [16, 17]. It has been shown that the overexpression of neurotrypsin in mice results in precocious sarcopenia due to the degeneration of NMJs [17]. Furthermore, elevated plasma levels of a 22 kDa C‐terminal Agrin fragment (CAF), which is cleaved by neurotrypsin, are observed in older adults, and CAF has been proposed to be a potential marker for sarcopenia [18–20].

Agrin is expressed in both motor neurons and skeletal muscles [21, 22]. Nevertheless, the isoform of Agrin expressed in skeletal muscle proves ineffective in inducing the aggregation of AChR due to the absence of the Lrp4 binding domain, critical for MuSK phosphorylation stimulation [14, 15]. Muscle Agrin binds to α-dystroglycan and interacts with membrane laminins, indicating a role in linking the extracellular matrix (ECM) to the cytoskeleton [23–25]. Overexpression of a miniaturized form of Agrin could retain basal laminins and alleviate dystrophic symptoms in a mouse model of congenital muscular dystrophy (CMD) [26–28]. However, the functional loss of Agrin in skeletal muscle remains poorly understood.

Here, we observed that the expression of Agrin in skeletal muscle is surprisingly reduced in aged mice, suggesting a potential role for Agrin in muscle aging. Furthermore, the conditional deletion of Agrin in skeletal muscle accelerated the onset of sarcopenia. Conversely, increasing Agrin levels in skeletal muscle improved muscle capacities and mitigated myofiber atrophy in aged mice. Mechanistically, the enhancement of Agrin improved muscles through the stimulation of Yap signaling and the expression of dystroglycan. Therefore, muscle Agrin emerges as a contributor to the pathogenesis of age-related sarcopenia, and suggest Agrin intervention in muscle holds promise as a novel therapeutic strategy for sarcopenia.

## Materials and methods

### Mouse strains

*Agrin* ^*f/f*^ mice were described previously [29, 30]. *Pax7-Cre* mice were purchased from the Jackson Laboratory (stock # 017763). All mice were maintained on a C57BL/6J genetic background, housed in a room with a 12 h light/dark cycle and had free access to water and standard rodent chow diet. Experimental procedures were approved by The Research Ethics Committee of Nanchang University-BioMedicine.

### Grip strength measurement

Grip strength was measured using a digital grip strength meter (Columbus Instruments) following the manufacturer’s instructions. Briefly, mice were allowed to grip a square metal grid that was connected to the hanging scale with their forelimbs. With their hind limbs suspended, mice were gently pulled horizontally by the tail until the grip was released.

### Treadmill test

The treadmill running distance test was performed using an Exer-3/6 treadmill (Columbus Instruments) as described previously [31]. Briefly, after three consecutive days of training, mice were placed on the track and started at an initial running speed of 10 m/min for 5 min, and speed was gradually increased by 2 m/min every 2 min until exhaustion. Exhaustion was determined when the mouse was unable to run on the treadmill for 10 s after five electric shocks. The total running distance was recorded by Treadmill software (Columbus Instruments).

### In vivo twitch and tetanic force measurement

Muscle twitch and tetanic force analyses were performed as previously reported [31, 32]. Briefly, mice were anesthetized with isoflurane continuously supplied by a VetFlo anesthesia system (Kent Scientific) and placed on a 37 °C heating pad. With gentle pressing via knee clamps, the left feet were fixed onto a footplate that was connected to a servomotor (Aurora Scientific 1300 A). The ideal position for muscle contraction was found by adjusting the distance between the footplate and the knee and measuring muscle force by stimulating the nerve with a single electrical stimulation (100 mA current, 0.2 ms pulse width). When muscle force no longer increased, the position was considered ideal for muscle contraction. To determine the best stimulation strength, single-muscle electrical stimulation began starting at a current of 100 mA and a pulse width of 0.2 ms in the ideal position, and muscle force was measured at every 30 mA increase, with an interval of 30 s. When muscle force no longer increased, the current was considered the ideal stimulation strength. In the ideal position and at the ideal stimulation strength, twitch force was measured by stimulating the muscle with a single electrical stimulation (0.2 ms pulse width), which was repeated ten times at an interval of 30 s. The tetanic force was measured by stimulating the muscle with a duration of 300 ms and a pulse width of 0.2 ms at a series of frequencies from 50 to 125 Hz (50, 80, 100 and 125 Hz) and an interval of 2 min. Twitch and tetanic forces were normalized to body weight.

### Hematoxylin and eosin staining

As described previously [31, 33], cryosections from freshly frozen tissues were washed with PBS 3 times, incubated in hematoxylin (#51275, Sigma) for 15 min, rinsed with deionized water and washed with tap water for at least 5 min to allow the stain to develop. Slides were then incubated in eosin (#R03040, Sigma) for 3 min. The slides were dehydrated sequentially with 75%, 95 and 100% ethanol for 2–5 min each. Slides were finally placed in xylene for 20 min and mounted with cytoseal. Images were taken with an Olympus FSX-100 camera, and image analysis was conducted using National Institutes of Health (NIH) ImageJ software.

### Western blot and analysis

Muscles were lysed in modified RIPA buffer (#C500005, Sangon Biotech) and protease inhibitors, including 1 mm phenylmethylsulfonyl fluoride, 1 μg/μl pepstatin, 1 μg/μl leupeptin and 2 μg/ml aprotinin. After centrifugation at 10000 RPM at 4 °C, the supernatant (lysate) was collected. Protein concentration was measured using a Pierce BCA kit (#23225, Thermo Scientific). Samples (50 μg protein) were resolved by SDS‒PAGE and transferred to a nitrocellulose membrane, which was incubated in 5% milk in phosphate buffered saline (PBS)-0.3% Tween 20(#A600560, Sangon Biotech) overnight at 4 °C and then with the following primary antibodies in 2% milk in PBS-Tween buffer: anti-MuSK (1:1000, sc-33204, Santa Cruz Biotechnology), anti-YAP (1:1000, #4912, Cell Signaling Technology), anti-YAP^ser127^ (1:500, #4911, Cell Signaling Technology), anti-Alpha Dystroglycan (1:500, ab106110, Abcam) and anti-GAPDH (1:2000, #437000, Thermo Scientific). After washing, the membrane was incubated with PBS-Tween buffer containing HRP-conjugated goat anti-mouse and anti-rabbit IgG from Pierce (1:2000, PI-32230 (anti-mouse), PI-32260 (anti-rabbit)). The immunoreactive bands were exposed to autoradiography films, and the grayscale was quantified by ImageJ (NIH), as described previously [31, 32].

### Immunoprecipitation

Immunoprecipitation (IP) experiments were performed as described previously [31, 32]. Briefly, muscle tissues were homogenized and lysed in IP buffer (150 mM NaCl, 2.5 mM EDTA, 50 mM Tris–HCl, pH 7.4, 50 mM NaF, 2% SDS, 0.5% sodium deoxycholate, 20% glycerol, 0.1% sodium vanadate, 1% PMSF). Then, the lysate was centrifuged at maximum speed (14,000 rpm) for 15 min at 4 °C. The supernatant was transferred to a new EP tube, 100–200 μg protein lysates were taken, an appropriate volume of 4× sample buffer was added, and the mixture was boiled at 95 °C for 5–10 min (used as input). The remaining supernatant was combined with 500–1000 µl (approximately 500–1000 µg protein) lysates, and 1–2 μg IP antibody was added overnight at 4 °C with rotation. Afterward, 5 μl protein A/G agarose beads were added to the lysates (#20399, Pierce) for 2–3 h at 4 °C. Finally, the protein was pulled down with beads, 20–30 μl of the appropriate 2× sample buffer was added, and the mixture was boiled at 95 °C for 5–10 min. The supernatant was collected and evaluated by SDS-PAGE.

### Immunostaining

For NMJ analysis, the TA muscle fibers were fixed in 4% formaldehyde overnight and permeabilized for 2 h with 0.5% Triton X-100(#HFH10, Thermo Scientific) in 3% BSA(#A602449, Sangon Biotech) and 3% goat serum (#16210064, Thermo Scientific). Samples were then incubated with a mixture of CF568-conjugated a-bungarotoxin (1:2000, Thermo Scientific) and antibodies neurofilament (NF) (1:1000, ab7795, Millipore) and synaptophysin (1:500, DAK-SYNAP, DAKO) at 4 °C overnight. After washing with PBS three times for 1 h each, muscle fibers were incubated with goat anti-mouse/anti-rabbit IgG conjugated with Alexa Fluor-488 (1:1500, Thermo Scientific) overnight at 4 °C.

For muscle and SC analysis, after being fixed in 4% formaldehide overnight, single muscle fibers or muscle sections were blocked for 30 min in a blocking buffer containing 5% goat serum, 2% BSA, 0.2% Triton X-100 and 0.1% sodium azide in PBS. Samples were then incubated with the following primary antibodies diluted in blocking buffer for 1 h at room temperature: anti-Agrin (1:200, D2, Santa Cruz Biotechnology), anti-α-Laminin (1:1000, L9393, Sigma), anti-Ki67 (1:1000, ab15580, Abcam) anti-MyoD (1:200, sc-304, Santa Cruz Biotechnology), anti-PAX7 (1:50, PAX7, Developmental Studies Hybridoma Bank (DSHB), anti-MyoG(1:50,F5D, DSHB), anti-eMyHC(1:100, F1.652, DSHB) and anti-MHC type2b (1:50, BF-F3, DSHB). Samples were then incubated with secondary antibodies and DAPI for 30 min at room temperature and mounted with fluorescent mounting media (#S36937, Thermo Scientific). Images were collected with an Olympus FSX-100 camera, and image analysis was conducted using National Institutes of Health (NIH) ImageJ software.

### Cardiotoxin injection

Muscle regeneration was induced by injecting 50 μL CTX (#217503, 10 μM, Sigma) into the mid-belly of TA muscles. Muscles were harvested at different time points after injection and fixed in 4% formaldehyde, submerged in 30% sucrose, embedded in OCT compound (Sakura Finetek), and quickly frozen in dry ice-cooled isopentane. Muscles were cut into 10-μm-thick sections for staining.

### AAV9 production and animal treatment

The PXX-CB-mini-agrin plasmid was a gift from Dr. Chunping Qiao (University of North Carolina at Chapel Hill, NC, USA). As described previously [28, 34], the mouse mini-agrin cDNA (GenBank accession no. AY914875) was obtained from the total RNA from the kidney. A signal peptide sequence was added in front of the N-terminus to increase protein secretion levels. The miniagrin cDNA was then cloned into an AAV vector plasmid under the transcriptional control of CMV enhancer and chicken β-actin promoter (CB promoter).

AAV9-CB-mini-agrin and AAV9-Control virus were prepared by OBiO Technology (Shanghai, China). The virus (30–50 μl of 2 × 10^12^–1 × 10^13^ vg/ml) was delivered by intramuscular injection or by intravenous injection (via the tail vein) (5 × 10^11^–1 × 10^12^ vg per mouse).

### Primary myoblast culture and viral infection of myoblasts

As described previously [33], primary myoblasts were isolated from fore and hind limb skeletal muscle. Muscles were minced and digested in a mixture of type I collagenase and dispase B (Roche Applied Science). Cells were then filtered from debris, centrifuged and cultured F-10 Ham’s medium (Thermo Scientific), supplemented with 20% fetal bovine serum, 4 ng/ml basic fibroblast growth factor and 1% penicillin-streptomycin (Thermo Scientific) on collagen-coated dishes.

AAV9-CB-mini-agrin and AAV9-Control virus were infected to 10-cm plates of aged myoblasts from 18–20 months old mice. After 6 h, fresh Ham’s complete medium was added to replace the virus solution and samples were collected after an additional 72 h.

### qRT‒PCR

Total RNA of mouse muscles was extracted using TRIzol reagent (Sigma) and transcribed into cDNA templates using High-Capacity cDNA Reverse Transcription Kits (Sigma) following the manufacturer’s instructions. Quantitative PCRs were run on a StepOne PlusTM Real-Time System (ABI System) using PowerUpTM SYBR Green Master Mix (Thermo Scientific). The primers for specific genes are listed in Supplementary Table S1.

### Statistical analysis

Data were assumed to be normally distributed and to have equal variance between groups. Randomization was not performed. Datasets were statistically compared using one- or two-way analysis of variance. Two-tailed, unpaired t tests were used to compare data between two groups. Unless otherwise indicated, data are expressed as the mean or mean ± standard error of the mean (SEM). All analyses were performed in a double-blinded manner to genotype.

## Results

### Agrin expression levels were decreased in aged muscles

**Fig. 1:**
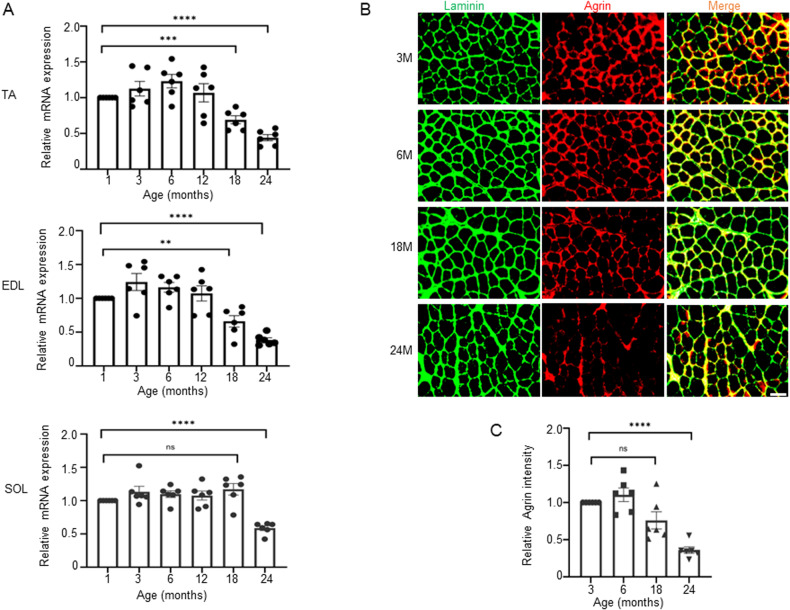
Reduced Agrin levels in aged muscles of mice. **A** qRT‒PCR of *Agrin* in TA, EDL and soleus muscles from mice at different ages. *n* = 6 mice. **B** Representative immunofluorescence staining of α-Laminin (green) and Agrin (red) in TA muscles from WT mice at 3, 6, 12 and 24 months of age. Scale bar = 50 μm. **C** Quantification of data presented in **B**, *n* = 6 mice, **p < 0.01, ****p < 0.0001.

To assess the contribution of Agrin to the maintenance of skeletal muscle function, we initially examined the mRNA and protein levels of Agrin in the skeletal muscles of aging mice. Surprisingly, in contrast to the elevated serum Agrin fragments observed in patients with sarcopenia, the expression of Agrin was found to be decreased in the skeletal muscles of aged mice. In Fig. 1A, it is evident that the mRNA level of Agrin remained unchanged in adult muscles (2 to 12 months of age) but exhibited a significant decrease in aged muscles (24 months of age), encompassing the tibialis anterior (TA), extensor digitorum longus (EDL), and soleus (SOL) muscles. Notably, at 18 months of age, a reduction in Agrin was observed in the fast-twitch (TA and EDL) muscles, but not in the slow-twitch soleus muscle (Fig. 1A), suggesting a preferential decrease of Agrin in fast-twitch muscles. Subsequently, through immunohistochemistry staining of muscle cross sections, we further confirmed that the protein level of muscle Agrin declined during aging (Fig. 1B, C). These results collectively underscore the close association between aging and the expression of skeletal muscle Agrin. The age-dependent reduction in muscle Agrin, observed at both mRNA and protein levels, prompted us to investigate the consequences of Agrin depletion in mice to gain deeper insights into the role of Agrin in muscle homeostasis and function during aging.

### Ablation of Agrin in skeletal muscle accelerated muscle aging

**Fig. 2:**
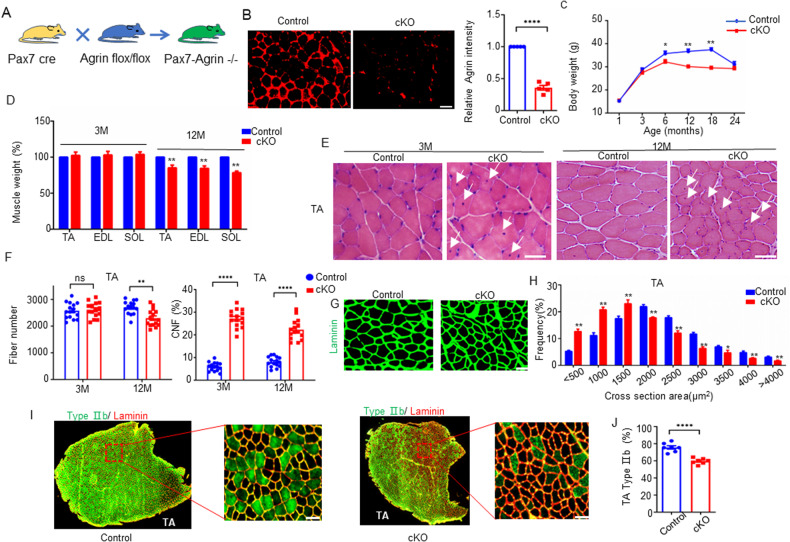
The absence of Agrin leads to progressive muscle fiber atrophy, fiber type conversion, and loss of muscle mass. **A** Schematic diagrams of *Pax7*-Cre-driven deletion of Agrin in mice. **B** Representative immunofluorescence staining of Agrin (red) in control and cKO TA muscle sections and quantification of data, *n* = 5 mice per genotype. **C** Bodyweights of control and cKO mice at different ages. *n* = 15 mice per genotype. **D** Muscle weights of control and cKO mice at 3 and 12 months of age. *n* = 15 mice per group. **E** Representative H&E staining images of TA muscle (fast twitch) cross-sections. **F** Quantification of total fiber number and CNF percentage in TA muscles (fast twitch). *n* = 15 mice per group. **G** α-Laminin staining showing the relative size of myofibers in TA muscles from mice at 12 months of age. **H** Frequency of distribution for CSA (µm^2^) of TA muscle (*n* = 5 per genotype). **I** Representative immunofluorescence staining of α-Laminin (red) and MyHC-2B (green) in TA muscle in mice at 12 months of age. **J** Quantification of data in **I**, *n* = 7 mice per genotype. Scale bar = 50 μm, *p < 0.05, **p < 0.01, ****p < 0.0001.

Agrin is a secreted protein, and Agrin-knockout mice (*Agrin*−/−) are born lethal due to an inability to form NMJs [35]. *Pax7* Cre is a mouse strain in which the *Pax7* promoter drives the expression of Cre in progenitor cells of skeletal muscle lineage and satellite cells (SCs) [36, 37]. Here, to further reveal the role of Agrin in skeletal muscle while avoiding the influence of paracrine Agrin, we crossed *Agrin* LoxP/LoxP mice with *Pax7* Cre mice to obtain conditional *Agrin*-knockout (cKO) mice in the progenitor cells of skeletal muscle and their descendent SCs and myofibers (Fig. 2A, Supplementary Fig. S1). Immunohistochemical analysis confirmed the loss of Agrin protein expression in the skeletal muscles of the cKO mice (Fig. 2B). cKO mouse weights were not significantly reduced compared to those of *Agrin* ^*f/f*^ mice (control) at 1 and 3 months of age, but they were significantly reduced at 6, 12, and 18 months of age. However, this difference disappeared at 24 months of age, perhaps because of the weight loss observed in control mice (Fig. 2C). Weight loss in cKO mice may be attributed to a reduction in skeletal muscle mass. To investigate this, we measured skeletal muscle weights in both cKO and WT mice. As anticipated, a decrease in skeletal muscle weights was evident in cKO mice at 12 months of age. However, no discernible differences were observed at 3 months of age compared to control mice (Fig. 2D). These findings suggest that the loss of muscle mass might be, at least in part, accountable for the weight loss observed in cKO mice.

We next investigated the consequences of Agrin deficiency on skeletal muscle fibers. TA muscles were stained with hematoxylin and eosin (H&E) and are shown in Fig. 2E. Quantitatively, the percentage of central nuclei fiber (CNF), an indicator of muscle regeneration, was increased in the muscles of cKO mice compared to those of control mice at both 3 months and 12 months of age. Moreover, the number of total muscle fibers was not different at 3 months but was reduced by 12.0% (2613 ± 73.1 vs. 2299 ± 82.3, *n* = 15) at 12 months of age in cKO mice (Fig. 2F). Additionally, at 12 months of age, in addition to the reduction in the number of fibers, cKO muscles possessed a larger number of small-diameter myofibers (cross section area <1500 µm^2^) than the muscles of control mice (Fig. 2G and H). The switching of muscle fiber type composition, with a decrease in the proportion of fast-twitch fiber types, is a hallmark of muscle aging [2, 38, 39]. Here, we also quantified fiber type composition in the muscles of cKO and control mice. Notably, at 12 months of age, the proportion of type 2B fast twitch fibers was decreased by 21.2% in cKO mice compared with that in control mice (Fig. 2I, J). Altogether, these results demonstrate that loss of Agrin in skeletal muscle accelerates muscle atrophy and aging and that Agrin is necessary to maintain normal muscle fibers.

### Grip strength and contractile capacity were impaired in Agrin cKO mice

**Fig. 3:**
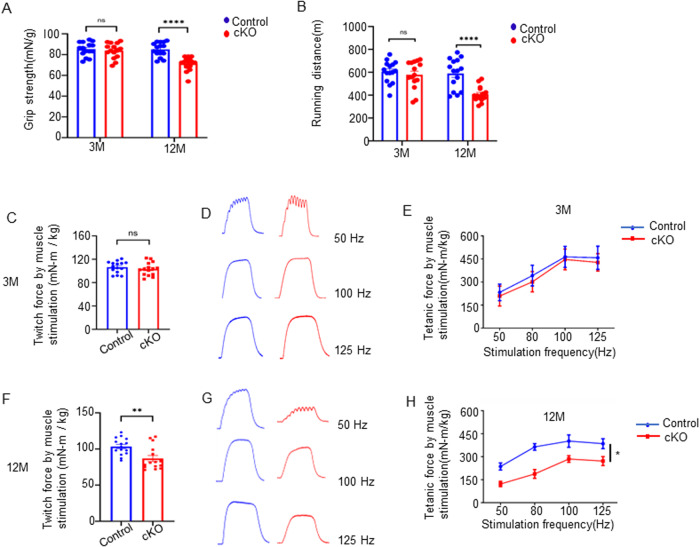
The absence of Agrin leads to progressive muscle function deficits. Grip strength (**A**) and running distance (**B**) of control and cKO mice at 3 and 12 months of age. *n* = 15 mice per group. Comparable single twitch and tetanic forces between control and cKO mice at 3 months (**C**–**E**) and 12 months (**F**–**H**) of age. **D**, **G** Representative tetanic curve. *n* = 15 mice per group. *p < 0.05, **p < 0.01, ****p < 0.0001.

We next determined whether Agrin deficiency affected skeletal muscle function. First, we measured the grip strength of cKO and *Agrin*^*f/f*^ control mice. Remarkably, cKO mice had significantly reduced grip strength at 12 months of age compared with control mice (85.1 ± 1.8 vs 71.4 ± 1.7, *p* < 0.0001, *n* = 15) (Fig. 3A). Accordingly, at this age, the running time for the treadmill test was decreased by 36.4% in cKO mice compared to control mice (589.4 ± 34.1 vs 407.7 ± 17.1, *p* < 0.0001, *n* = 15) (Fig. 3B). Next, we performed in situ contractility tests by evaluating muscle strength following electric stimulation of TA muscles. Compared with control mice at 12 months of age, muscles in cKO mice displayed lower twitch and tetanic contractile capacities, but there was no difference at 3 months of age (Fig. 3C–H). Together, these in vivo results indicate that cKO mice exhibit weaker strength and experience earlier progression of age-associated declines in muscle function.

### NMJ structure was normal in Agrin cKO mice

**Fig. 4:**
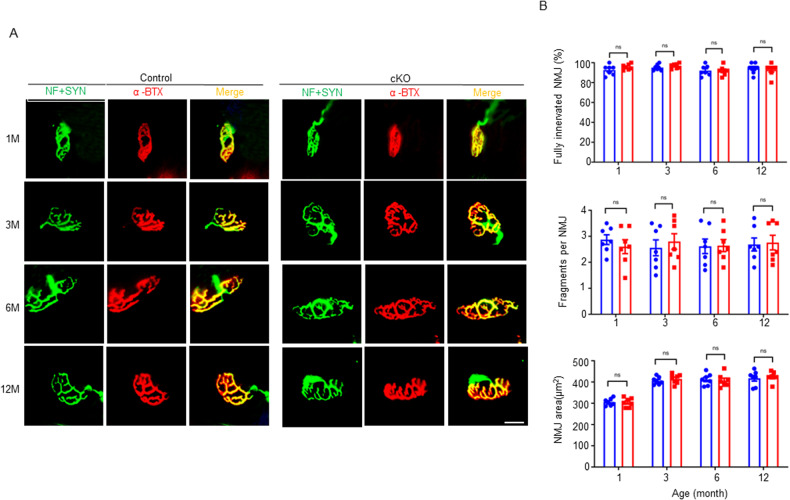
NMJ structure was normal in Agrin cKO mice. **A** Representative images of NMJs in cKO and control mice at 1, 3, 6 and 12 months of age. TA muscles were stained with α-BTX to label AChRs (red) and NF/SYN (green) to label nerve terminals. Scale bar = 10 μm. (**B**) Quantification of data in **A**. *n* = 8 mice per group and 25–30 NMJs of each mouse were counted.

To determine whether NMJ structure was altered in Agrin cKO mice, TA muscles were stained with antibodies against neurofilament (NF) and synaptophysin (SYN) to visualize axons and nerve terminals and with CF568-conjugated α-bungarotoxin (RBTX) to visualize AChRs [31]. As shown in Fig. 4A, NMJs were normal in both cKO and control mice, with most NMJs being fully innervated and continuous, and there were no differences in the areas of the AChR clusters (Fig. 4B).

### Muscle satellite cells declined in Agrin cKO mice

**Fig. 5:**
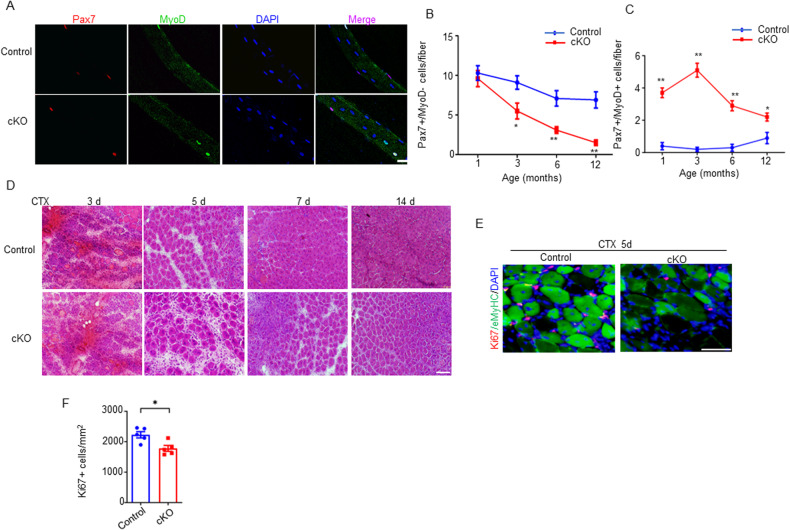
The absence of Agrin disrupted muscle stem cells and impaired muscle regeneration. **A** Representative immunofluorescence staining of Pax7 (red) and MyoD (green) for control and cKO EDL single muscle fibers. Scale bar = 10 μm. **B**, **C** Quantitative data of quiescent (Pax7+ MyoD−) and activated (Pax7− MyoD+) SCs in EDL single muscle fibers from control and cKO mice. *n* = 5 mice per group, 20 myofibers per mouse. **D** Representative H&E staining images of CTX-induced injured TA muscles from control and cKO mice. Scale bar = 100 μm. **E** Representative immunofluorescence staining of eMyHC (green) and Ki67 (red) in TA muscle sections after CTX injection. Scale bar = 50 μm (**F**) Quantification of data in **E**, *n* = 5 mice per genotype. *p < 0.05, **p < 0.01, ***p < 0.001.

In addition to a gradual loss of muscle mass and function, muscle aging is characterized by a decline in regenerative capacity [2, 40]. The regenerative capacity of skeletal muscle relies on SCs, a population of muscle stem cells adjacent to the plasma membranes of myofibers. Numerically and functionally compromised SCs have been observed in aged mice and humans. In the context of the weaker strength and prematurely aging muscle fibers observed in Agrin cKO mice, we hypothesized that the number of SCs decreased and repair capacity declined. To test this hypothesis, we first determined the number of SCs. Fresh single muscle fibers were isolated from the EDL muscles of cKO and wild-type control mice and stained with Pax7, Myf5, MyoD and MyoG antibodies to label quiescent and activated SCs [41]. Interestingly, compared with the control muscle, the cKO muscle had a similar number of quiescent SCs (Pax7+/MyoD−) at the early stage of 1 month of age, but then the number decreased rapidly and significantly at 3 months of age (Fig. 5A, B). In contrast, the number of activated SCs (Pax7+/MyoD+) was increased in the muscles of cKO mice as early as 1 month of age (Fig. 5C). Moreover, the number of Myf5+ cell was also reduced while MyoG+ cell increased in cKO mice (Supplementary Fig. S2). These results demonstrate that SC quiescence is disrupted in Agrin cKO mice.

We next employed the cardiotoxin (CTX)-induced muscle injury regeneration model to investigate the in vivo regenerative capacity of muscle with or without Agrin. Notably, in this set of experiments, we selected 3-month-old control and cKO mice. At this timepoint, muscle atrophy and functional defects had not yet appeared. As shown in Fig. 5D, at day 3 postinjury, a similar degree of damage was observed in the TA muscle sections from *Agrin* ^*f/f*^ control and cKO mice, manifested by many infiltrating immune cells and denatured muscle fibers. At days 5 and 7 postinjury, many small regenerating muscle fibers (central nuclei fibers) appeared in the muscle sections of the control mice. Such regenerating muscle fibers were significantly reduced in the muscles of Agrin cKO mice. At day 14 postinjury, the injured TA muscles from control mice were almost completely regenerated, but those from Agrin cKO mice remained largely unrepaired, suggesting that the muscle regeneration was delayed in Agrin deficient mice.

The delay of muscle regeneration may also be caused by the low proliferation ability of muscle SCs in the process of muscle injury repair. To assess this condition, muscle sections from mice at day 5 postinjury were stained with Ki67, a marker of proliferating cells. As shown in Fig. 5E, F, the number of Ki67+ proliferating cells was significantly decreased in the muscles from cKO mice, indicating that the proliferation of SCs was inhibited. Altogether, these results reveal that muscle SCs are disrupted and regenerative capacity is diminished in Agrin cKO mice. These effects occurred prior to the appearance of muscle fiber atrophy and weakness. The delay of regeneration after muscle injury in cKO mice was attributed to both the decreased number of quiescent SCs and the diminished proliferative capacity of SCs during muscle injury repair.

### Increasing Agrin in skeletal muscle reversed age-associated sarcopenia in mice

**Fig. 6:**
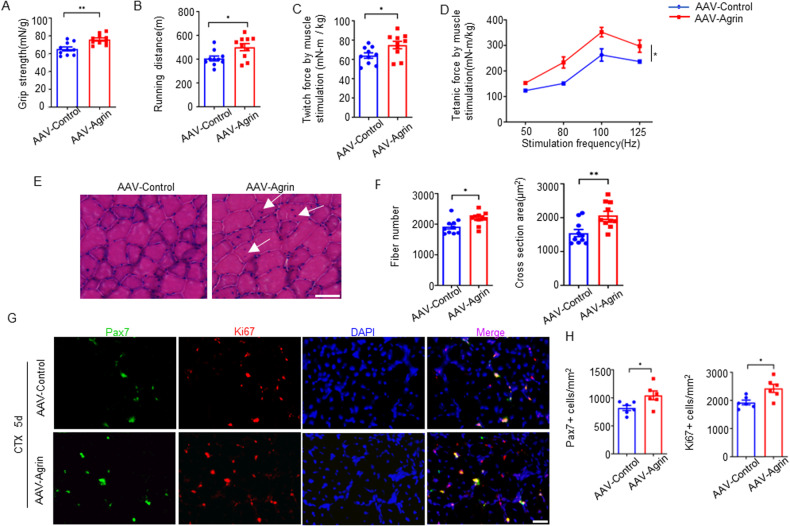
AAV-mini-Agrin reversed age-associated muscle weakness. Grip strength (**A**), running distance (**B**), single twitch force (**C**) and tetanic force (**D**) of control and AAV-Agrin treated mice (5 weeks after administration). *n* = 10 mice per group. **E** Representative H&E staining images of TA muscle sections from control and AAV-Agrin treated mice. Scale bar = 50 μm. **F** Quantification of the total number and average cross-sectional area. *n* = 10 mice per group. **G** Representative immunofluorescence staining of Pax7 and Ki67 in TA muscle sections from control and AAV-Agrin treated mice 5days after CTX injected. Scale bar = 20 μm. **H** Quantification of data in **G**. *n* = 6 mice per group, 15–20 section images per mouse. *p < 0.05, **p < 0.01.

In light of the observations that Agrin levels decreased during aging and that loss of Agrin in skeletal muscle caused earlier onset of sarcopenia-like muscle deficits, we hypothesized that insufficient Agrin might be a mechanism of age-associated sarcopenia. This hypothesis predicts that increasing Agrin expression could diminish sarcopenia-like deficits in aged muscles. To test this hypothesis, we produced an adeno-associated virus expressing mouse mini-agrin that contains an amino-terminal domain, the first follistatin-like domain, and the α-dystroglycan binding domain but no Lrp4 binding domain (Z8^−^) of mouse Agrin [26]. It has been proven that this miniature version of Agrin could be increased expression in muscles by direct intramuscular injection and render remarkable therapeutic benefits in a mouse model of congenital muscular dystrophy [28, 34]. Here, TA and gastrocnemius muscles from 22- to 24-month-old mice were injected with AAV9- CB-mini-Agrin and AAV9-control. As expected, grip strength, running distance, and contractile force increased 5 weeks after AAV-Agrin injection (Fig. 6A–D). Accordingly, compared to AAV-control-injected aged mice, the number and diameter of muscle fibers were also increased in AAV-Agrin-injected aged mice (Fig. 6E, F), suggesting that AAV-Agrin reduced the number of atrophic muscle fibers in aged mice. Moreover, AAV-Agrin treatment of CTX-injected aged mice increased the number of SCs and their proliferative capacity, which was revealed by increased numbers of Pax7-positive and Ki67-positive cells (Fig. 6G, H). Together, these results suggest that increased expression of Agrin may prevent skeletal muscle from wasting and weakening during aging.

### Yap signaling and α-DG were involved in Agrin deficiency and sarcopenic muscles

**Fig. 7:**
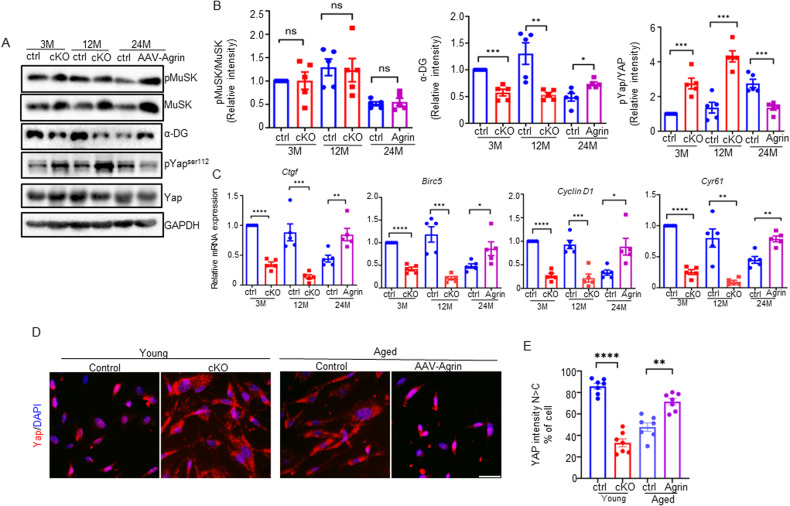
Yap signaling and α-DG were involved in Agrin deficiency and sarcopenic muscles. **A** Western blot analysis of Agrin mediated pathways in skeletal muscles from Agrin-deficient and aged mice. **B** Quantification of data in **A**. *n* = 3 mice per group. **C** qRT‒PCR analysis of Yap downstream targets in skeletal muscles from Agrin-deficient and aged mice. *n* = 5 mice per group. **D** Representative images of Yap (red) in young (2-3 months) and aged (18–20 months) myoblast. **E** Quantification of data in **D**, *N* > C refers to predominant nuclear YAP. *n* = 7 mice per group, Scale bar = 50 μm.*p < 0.05, **p < 0.01, ***p < 0.001, ****p < 0.0001.

Previous reports highlighted that Agrin could trigger various pathways, including Lrp4/MuSK, dystroglycans, and Hippo [14, 42–44]. These pathways are virtually also responsible for maintaining SC or skeletal muscle function [45–49]. To further investigate the underlying molecular mechanisms by which Agrin deficiency leads to sarcopenic myofibers, we examined these potentially Agrin dependent signaling pathways in muscle cells from cKO and aged mice. As shown in Fig. 7A, B, MuSK phosphorylation was reduced in muscle cells from aged mice (24 months) compared to young mice (3 months), consistent with previous reports. However, no significant changes in phospho-MuSK were observed in either cKO or aged mice treated with AAV-Agrin. Interestingly, the expression of α-dystroglycan (α-DG), a receptor of Agrin that plays a critical role in maintaining the integrity of the muscle membrane, was reduced in cKO and aged muscles but increased after AAV-Agrin treatment. In contrast, phosphorylation of Yap at serine 112, a key event in Yap regulation by the Hippo pathway, was significantly elevated in muscles from cKO mice at 3 months and 12 months of age. Moreover, the phospho-Yap ^Ser112^ level was increased in aged muscles but reduced after AAV-Agrin treatment (Fig. 7B).

The increase in Yap phosphorylation levels at serine 112 leads to the retention of Yap in the cytoplasm, thus affecting the expression of downstream genes [50, 51]. We next investigated whether the signaling genes downstream of Yap were involved. Quantitative real-time PCR showed that Yap targets (*CTGF, Birc5, CyclinD1, and Cyr61*) were downregulated in cKO and aged muscles but upregulated after AAV-Agrin treatment (Fig. 7C).

Next, to further investigate whether Agrin affected the balance between nuclear import and export of Yap in vitro. Primary myoblast were isolated and cultured at a low (10,000 cells/cm^2^) density, as high-density conditions will result in cells predominantly exhibiting cytoplasmic localization of Yap [52, 53]. As shown in Fig. 7F, consistent with increased Yap phosphorylation, Yap was shifted from the nucleus to the cytoplasm in Agrin cKO and aged muscle cells. Importantly, the nuclear localization of Yap was restored in AAV- Agrin infected cells (Fig. 7D, E). Together, these results suggest that Yap signaling and α-DG, rather than MuSK signaling, are involved in the accelerated sarcopenia onset caused by Agrin deficiency.

## Discussion

Neural Agrin is known for its indispensable role in formation and maintenance of NMJs. However, our current investigation reveals a novel aspect by highlighting the contribution of skeletal muscle-derived Agrin, rather than that from motor neurons, in the context of sarcopenia. Firstly, we demonstrated a decline in Agrin levels within skeletal muscles during the aging process. Secondly, the conditional loss of Agrin in the progenitors of muscle and SCs not only compromised muscle fiber size but also impeded muscle contraction ability, concurrently disturbing the quiescent state of SCs. Finally, elevating Agrin levels proved efficacious in ameliorating both muscle fiber morphology and function in aged mice. These findings unveil a mechanism underpinning muscle deterioration in the aging process and posit Agrin as a potential therapeutic target for addressing sarcopenia.

Recently, the C-terminal Agrin fragment (CAF) has emerged as a promising biomarker for neuromuscular disorders attributed to NMJ degeneration [18, 20]. Elevated CAF plasma levels have been observed not only in patients with sarcopenia but also in those with other muscle-wasting conditions, including diabetes, chronic heart failure, stroke, and cancer cachexia [20]. While these findings might suggest an increase in Agrin levels in sarcopenia patients, it is crucial to note that the measured CAF represents only a small fragment of Agrin cleaved by neurotrypsin, reflecting the activity of neurotrypsin rather than the overall expression of Agrin. Our results indicate a gradual decrease in Agrin expression during muscle aging, implying that muscle Agrin expression and plasma CAF levels are distinct and independent processes. An alternative explanation could be that elevated CAF levels may result from a compensatory response to Agrin deficiency in aged muscles. On the other hand, our findings align with the notion that Agrin expression diminishes in dystrophic muscles, corroborated by reduced Agrin expression observed in various mouse models of muscular dystrophy [54]. Additionally, we noted reports suggesting that neural activity induces muscle Agrin expression [55]. Consequently, the observed decrease in Agrin expression in dystrophic muscles may be attributed to insufficient innervation, although further investigation is required to confirm this hypothesis.

Our results also revealed that Agrin deficiency disrupted the quiescent state of SCs, leading to declines in stem cell function and number. SCs are a population of stem cells located in the basal lamina and plasma membranes of myofibers. The ECM in skeletal muscle provides the extracellular environment for SCs and is integral to SC activation, proliferation, and differentiation [40, 56, 57]. Our results present the initial evidence highlighting the significance of Agrin, an ECM component, in the maintenance of SCs. Interestingly, in Agrin-deficient mice, the number of quiescent SCs remained relatively unchanged at an early age (1 month) but exhibited a progressive decrease with advancing age. This suggests that the decline of SCs in Agrin-deficient mice is attributed to chronic depletion rather than acute reduction. However, it’s crucial to consider that this chronic depletion and decline in SCs might also stem from reduced α-dystroglycan (α-DG) levels and compromised sarcolemma integrity. This aspect requires further investigation in future studies. Additionally, due to limitations in effective antibodies and the utilization of *Pax7*-Cre mice, we were unable to ascertain whether the Agrin influencing SC maintenance originates from SCs or muscle fibers. More comprehensive investigations in the future are warranted to determine the expression of Agrin in SCs and elucidate the function of Agrin derived from SCs.

Throughout the aging process, the quiescent state of SCs undergoes disruption, leading to a decline in both their number and function [2, 40, 58]. However, the question of whether these declines in SCs are the cause or consequence of sarcopenia remains a subject of controversy. Brack et al. suggested that the loss of large fiber cell nuclei due to the decline in the number of SCs during aging is an important trigger for myofiber atrophy [59]. Nevertheless, Fry et al. showed that the reduced number of SCs and impaired muscle regeneration in sedentary mice neither accelerated nor exacerbated the development of sarcopenia [60]. Intriguingly, our results reveal that the number of quiescent SCs (Pax7+ and MyoD-) was reduced as early as 3 months of age in Agrin-deficient mice, occurring even before the onset of myofiber atrophy. This finding suggests that the disruption of the quiescent state and the reduction in the number of SCs act as triggering factors for the pathological phenotype of sarcopenia. This notion finds support in the work of Liu W et al., who demonstrated that the loss of SCs leads to muscle weakness, neuromuscular junction degeneration, and other symptoms associated with sarcopenia in mice [61].

Our study further highlights the favorable impact of Agrin during muscle aging. Prior research demonstrated that the overexpression of mini-Agrin effectively mitigates dystrophic phenotypes in a laminin-α2-deficient mouse model of congenital muscular dystrophy (CMD). Mechanistically, the authors proposed that Agrin acts as a crosslink between laminins and dystroglycans, thereby restoring the basal lamina and ameliorating dystrophic pathology [26]. Building upon these insights, our results reveal that the overexpression of mini-Agrin enhances the proliferative capacity of SCs during muscle regeneration in aged mice. Although our current study lacks in vitro validation, a recent report indicating that Agrin treatment promotes the proliferation of human myoblasts in aged donors lends support to our findings [62]. Collectively, these results suggest that Agrin not only contributes to the restoration of the basal lamina but also exerts a proliferative effect on SCs, introducing a novel mechanism by which Agrin ameliorates muscle atrophy. Investigating the impact of Agrin on muscle SCs in mouse models of CMD and other forms of muscular dystrophy would be of significant interest. Indeed, it has been shown that the expression of mini-Agrin significantly enhances muscle regenerative capacity in laminin-α2-deficient mice [27].

**Fig. 8:**
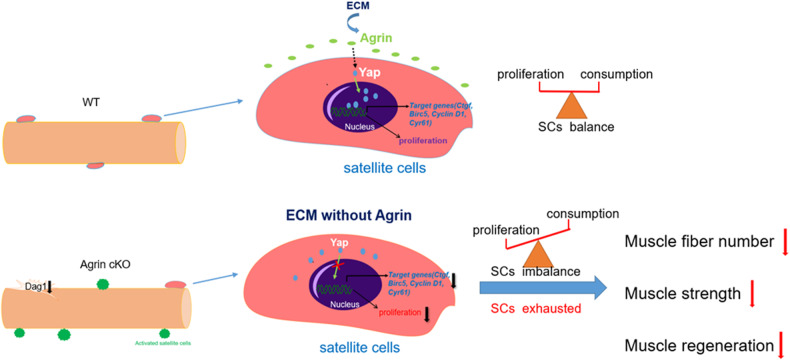
Proposed model: Agrin deficiency reduces Yap activity and dystroglycan expression, leading to SCs excessively consumption and exhaustion with age, eventually causing muscle weakness and premature muscle aging.

The Yap signaling pathway serves as a pivotal regulator of cell division and apoptosis, orchestrating organ growth across various species. Under normal conditions characterized by low Hippo signaling, Yap remains active and localized within the nucleus, modulating gene expression to govern cell proliferation and terminal differentiation [50, 53, 63]. It has been previously demonstrated that Yap activity, indicated by Yap^Ser112^ phosphorylation, is elevated in atrophic denervated muscle, amyotrophic lateral sclerosis model mice and Duchenne muscular dystrophy model mice [64, 65]. Moreover, Yap was reported to positively regulate SC proliferative dynamics in mice [48]. Our results unveil an upregulation of Yap activity in both aged and Agrin-deficient mice. Conversely, the overexpression of mini-Agrin in aged muscles leads to a downregulation of Yap activity, accompanied by an increase in the expression of downstream target genes. Consequently, SC proliferation is inhibited in Agrin-deficient mice with lower Yap activity, while it is promoted in Agrin-overexpressing mice with higher Yap activity. These findings collectively suggest that Agrin promotes SC proliferation through Yap nuclear translocation. We propose the hypothesis that under normal conditions, Agrin stimulates the translocation of Yap from the cytoplasm to the nucleus, ensuring the proper proliferation of muscle SCs and maintaining homeostasis. When Agrin is deleted or its expression is reduced (as in muscle-specific knockout or aged mice), the rate of Yap entry into the nucleus decreases, leading to a decline in the proliferative ability of muscle SCs. Conversely, Agrin deficiency reduces the expression of α-dystroglycan, resulting in the loss of fiber membrane integrity, perpetuating the continuous activation of muscle SCs. This excessive consumption and exhaustion of SCs with age eventually contribute to the onset of a sarcopenic phenotype, characterized by a reduced number of muscle fibers, decreased muscle strength, and impaired muscle regenerative ability (Fig. 8). Pursuing this hypothesis, it would be intriguing to experiment with increasing Yap activity in aging and Agrin cKO mice to observe if it mitigates sarcopenia phenotypes. However, it’s noteworthy that sustained activation of Yap in muscle could potentially have adverse effects on myoblast differentiation [48]. Therefore, maintaining a balanced level of Yap activity in muscle should be carefully considered in the future studies.

### Supplementary information

Supplemental Table and Figures

Original western blot1

Original western blot2

## Data Availability

The raw data supporting the findings of the current study are available from the corresponding author on reasonable request.
