# Supplementary material for: Deficiency of skeletal muscle Agrin contributes to the pathogenesis of age-related sarcopenia in mice
Source: Cell Death Dis. 2024 Mar 9;15(3):201. doi: 10.1038/s41419-024-06581-1 (PMC10925061; doi:10.1038/s41419-024-06581-1)
Supplement: Supplementary file 1 — Supplemental Table and Figures [file 41419_2024_6581_MOESM1_ESM.docx]

Table S1. Primer sequences used for qRT‒PCR

| Gene | Primer sequence |
| --- | --- |
| *Agrin* | F: 5’- GGACTCAGAAGGCTCCAACTGT-3’ |
|  | R: 5’- GTCAGCCATCTCTGGTGTGTAG -3’ |
| *Ctgf* | F: 5’- TGCGAAGCTGACCTGGAGGAAA -3’ |
|  | R: 5’- CCGCAGAACTTAGCCCTGTATG -3’ |
| *Birc5* | F: 5’- CCTACCGAGAACGAGCCTGATT -3’ |
|  | R: 5’- CCATCTGCTTCTTGACAGTGAGG -3’ |
| *Cyclin D1* | F: 5’- GCAGAAGGAGATTGTGCCATCC -3’ |
|  | R: 5’- AGGAAGCGGTCCAGGTAGTTCA -3’ |
| *Cyr61* | F: 5’- GTGAAGTGCGTCCTTGTGGACA -3’ |
|  | R: 5’- CTTGACACTGGAGCATCCTGCA -3’ |
| *GAPDH* | F: 5’-GTG AAG GTC GGT GTG AAC GG-3’ |
|  | R: 5’-CAA GCT TCC CAT TCT CGG CCT-3’ |


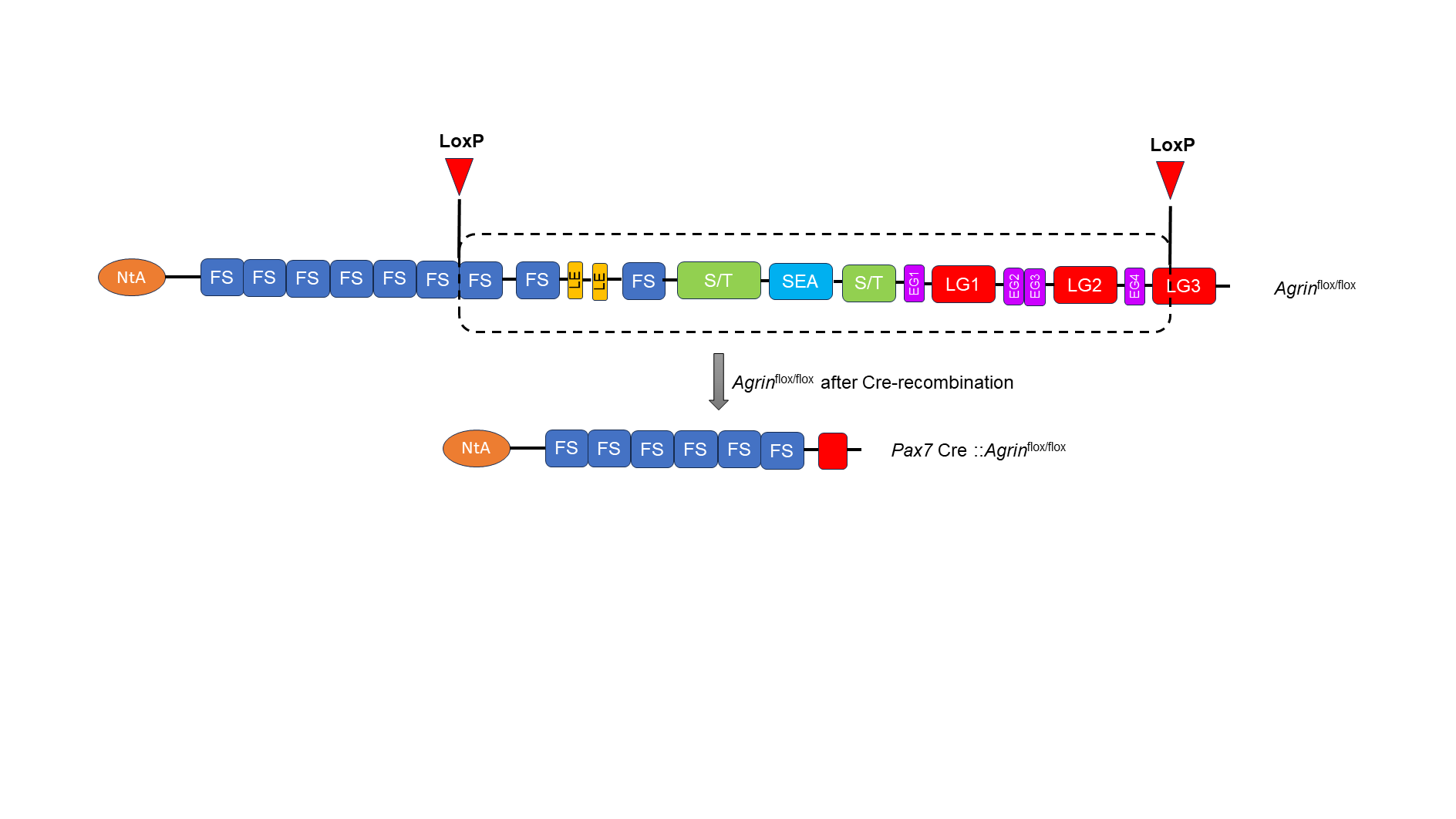


**Figure S1**. Schematic representation of mouse Agrin protein and properties of the mutant *Agrin* alleles. NtA: N-terminal domain of Agrin, FS: follistatin-like, LE: laminin EGF-like, S/T: serine/threonine-rich, SEA: sperm protein/enterokinase/agrin, EG: EGF-like, LG: Laminin globular domains


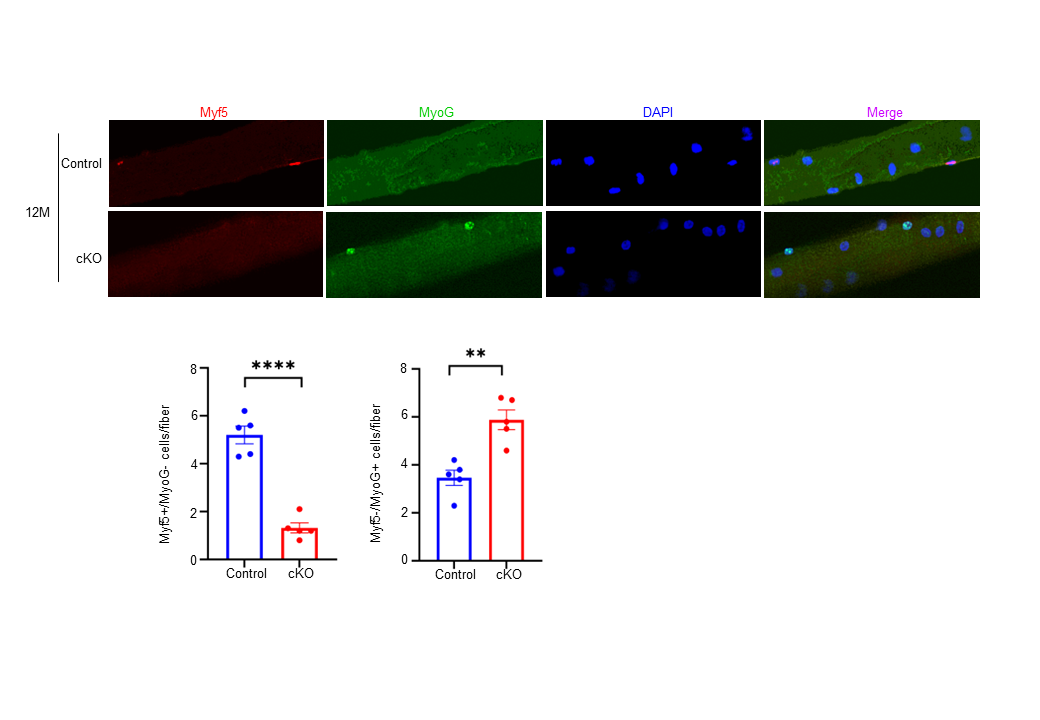


B

A

**Figure S2.** Satellite cells were over activated in Agrin cKO mice

(A). Representative immunofluorescence staining of Myf5 (red) and MyoG (green) for 12months old control and cKO EDL single muscle fibers. (B) Quantitative data of quiescent (Myf5+ MyoG-) and activated (Myf5- MyoG+) SCs in EDL single muscle fibers from control and cKO mice. n = 5 mice per group, 10 myofibers per mouse.
