# Supplementary figures and images for: Deficiency of skeletal muscle Agrin contributes to the pathogenesis of age-related sarcopenia in mice
Source: Cell Death Dis. 2024 Mar 9;15(3):201. doi: 10.1038/s41419-024-06581-1 (PMC10925061; doi:10.1038/s41419-024-06581-1)

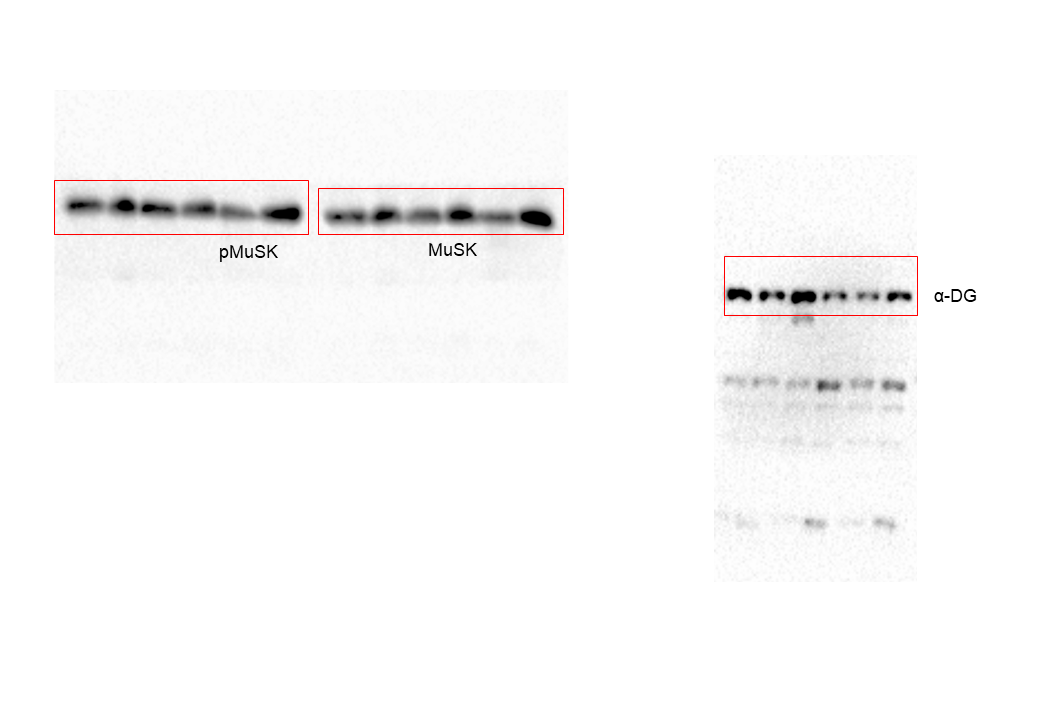

Supplement: Supplementary file 2 — Original western blot1 [file 41419_2024_6581_MOESM2_ESM.tif]

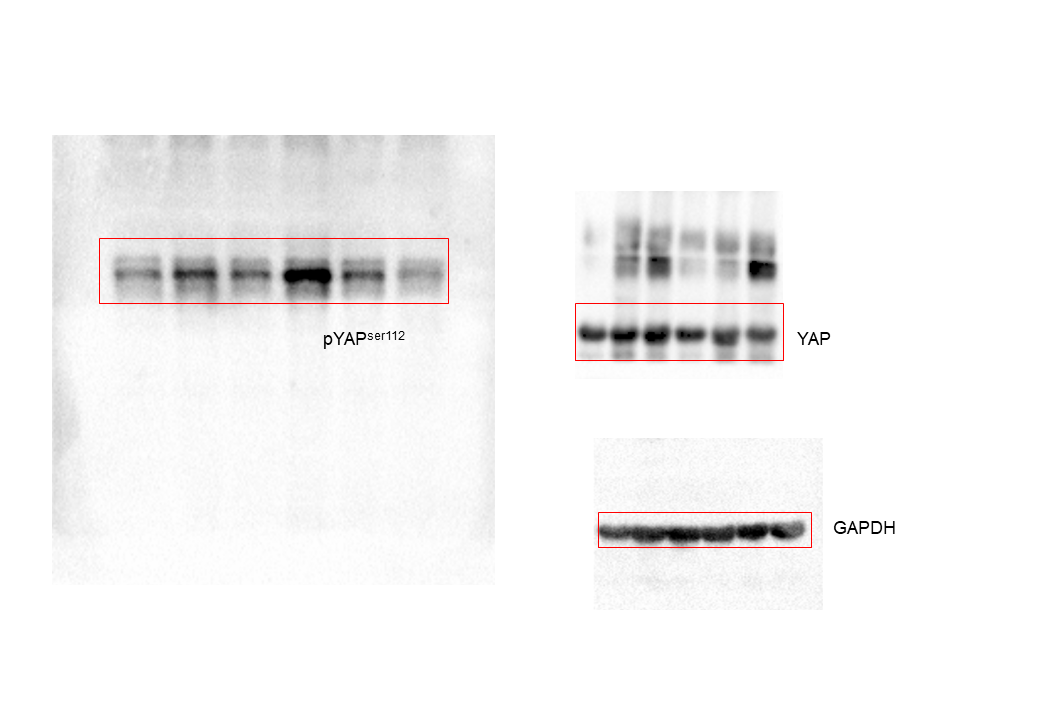

Supplement: Supplementary file 3 — Original western blot2 [file 41419_2024_6581_MOESM3_ESM.tif]
